# Introduction to the IX Meeting of the "Sociedade Brasileira de Biociências Nucleares". Caxambu, MG, Brazil, August 27-30, 2014

**DOI:** 10.1590/1414-431X20150000

**Published:** 2015-10-06

**Authors:** Silvia Maria Velasques de Oliveira

**Affiliations:** Comissão Nacional de Energia Nuclear, Instituto de Radioproteção e Dosimetria, Rio de Janeiro, RJ, Brasil

In the last two decades, the applications of ionizing and non-ionizing radiation in Biology and Medicine have been improved considerably in Brazil. The 2014 SBBN Congress, entitled "Framework, science and technology for preclinical and clinical studies with ionizing and non-ionizing radiation", was held in Caxambu, MG, during the XXIX FeSBE Annual Meeting. About 50 posters were presented in Radiobiology, Radiopharmaceuticals, Radiation protection, Biophotonics and Photobiology. For new radiopharmaceutical developments, the preclinical studies were essential because the "*in vivo*" biodistribution should be investigated by images before establishing the routine protocols. The clinical studies establish the absorbed doses in organs, by improving the compartimental models and allowing the prescription of the activities (amount of radiation) to be administered to humans. Both in preclinical and in clinical studies, quality control of radiopharmaceuticals labelling is one of the steps in the quality assurance system, which is mandatory for registry in the Health Regulatory Agency (ANVISA, in Brazil). The biological effects of ionizing radiation in Medicine were identified by biodosimetry in routine or in non-planned situations. In industrial applications, accidents have been reported. Treatments of radiological victims in different contexts, including reconstructive dosimetry for cutaneous radiation syndrome (CRS), should be obligatory disciplines in the professional curricula of the multidisciplinary teams. The low-intensity red and infrared laser effects are increasing in Regenerative Medicine, nevertheless the associated risks are unknown and there is a lack of safety requirements. Eight papers presented in the SBBN Congress were selected to be published in the Brazilian Journal of Medical and Biological Research, Volume 48(10), 2015.

2014 SBBN Congress President

